# The Long-term Outcome of Pediatric Kidney Transplantation in Iran: Results of a 25-year Single-Center Cohort Study

**Published:** 2017-05-01

**Authors:** G. Naderi, A. Latif, S. Karimi, F. Tabassomi, S. T. Esfahani

**Affiliations:** 1 Department of Kidney Transplantation, Dr. Shariati Hospital, Tehran University of Medical Sciences, Tehran, Iran; 2 Department of General Surgery, Dr. Shariati Hospital, Tehran University of Medical Sciences, Tehran, Iran; 3 Department of Internal Medicine, Dr. Shariati Hospital, Tehran University of Medical Sciences, Tehran, Iran; 4 Department of Pediatric Nephrology, Children’s Medical Center, Tehran University of Medical Sciences, Tehran, Iran

**Keywords:** Kidney transplantation, Kidney failure, chronic, Child, Pediatrics, Graft survival, Immunosuppression, Iran

## Abstract

**Background:**

Kidney transplantation is the optimal treatment for end-stage renal disease in children. However, long-term graft survival has not significantly improved among pediatric patients.

**Objective:**

To investigate the determinants of long-term graft survival among Iranian pediatric recipients of kidney transplantation.

**Methods:**

In a single-center cohort study, we studied 314 pediatric kidney transplantations performed from 1989 to 2013 at Dr. Shariati Hospital, Tehran, Iran. Different variables were collected for each patient and graft survival rates were calculated.

**Results:**

After a mean±SD follow-up period of 15.8±4.0 years, the mean±SD graft survival rate was 14.5±0.5 years; the 1-, 5-, 10-, and 20-year mean graft survival rates were 90%, 81%, 62%, and 62%, respectively. The corresponding patient survival rates were 100%, 99.4%, 97.8%, and 96.5%, respectively. Pre-emptive transplantation (p=0.006), and living graft donation (p=0.002) led to higher graft survival, while acute rejection (p=0.002), and primary disease of primary hyperoxaluria (p=0.001) led to lower graft survival. Chronic rejection was the most frequent cause of graft loss.

**Conclusion:**

Short-term graft survival still outpaces the long-term outcome. Modifying the mentioned determinants, with more intense immunosuppression for greater prevention of acute and chronic rejection, and increased rate of pre-emptive transplantation and living donor transplantation, long-term graft survival may significantly improve in future.

## INTRODUCTION

End-stage renal disease (ESRD) is an overwhelming illness especially in children. A recent study estimates the incidence of chronic kidney diseases (CKD) at 16.8 per million children population; the kidney transplantation rate in Iran is 7.2 per million children population [1]. Kidney transplantation is the optimal treatment for children with ESRD and offers substantial benefits including increased survival, and improved growth, social adjustment, and quality of life [2-5].

Recent advances in pre- and post-transplantation management, immunosuppressive medications, surgical techniques, and donor selection have contributed to improved patient and graft survival among pediatric kidney recipients [6-8]. Nonetheless, some previous studies stated that long-term graft survival rates have not significantly increased in this population, mainly because of infections, acute rejection episodes, and poor adherence to medications [6, 9-11]. It is important to identify the influencing factors of long-term kidney transplantation outcome among pediatric recipients. Single-center studies may confer more accurate information and longitudinal data on long-term consequences of kidney transplantation among pediatric patients [11, 12]. We therefore conducted this study to investigate the long-term outcome of kidney transplantation and its determinants among pediatric recipients in a 25-year cohort study.

## PATIENTS AND METHODS

This was a 25-year retrospective cohort study on pediatric patients who underwent kidney transplantation at Dr. Shariati Hospital, Tehran, Iran, between 1989 and 2013. All patients who aged 18 years or younger at the time of transplantation (n=297) were included in the study. A total of 17 patients underwent retransplantation that resulted in 314 pediatric kidney transplantation during the study period. All the graft nephrectomies were performed using the classical open extraperitoneal technique. This study was approved by the Medical Ethics Committee of Tehran University of Medical Sciences.

For each patient, data including age; sex; cause of ESRD; history of dialysis before transplantation; donor’s age, sex, and type; immunosuppressive regimen; presence of acute rejection and malignancy; cause of graft loss and mortality; and serum creatinine levels were obtained and analyzed to find independent factors of graft survival. Regarding the age, recipients and donors were categorized into age groups for easier analysis. In case of encountering a missing variable for a patient, the patient was excluded for that variable and the analysis was performed using other patients’ data.

The immunosuppressive regimen consisted of a triple combination of corticosteroid plus two other drugs based on the availability and applicability at the time of transplantation. Regarding the induction treatment, those patients who underwent transplantation after 1999 received thymoglobulin; others did not receive such treatment due to unavailability of the drug before 1999.

Acute rejection episode was defined as an abrupt increase in baseline serum creatinine level, and was biopsy-proven thereafter. The provided treatment for acute rejection consisted of 3-day high dose corticosteroids pulse therapy. In case of corticosteroid-resistance, thymoglobulin was administered. Graft loss was defined as absence of graft function due to patient death, graft injury using the RIFLE criteria, or the need to chronic dialysis and/or retransplantation. Therefore, graft survival was considered the period between the time of kidney transplantation and either graft loss, last date of follow-up with a functioning graft, or patient death. Meanwhile, patient survival was defined as the period between the time of kidney transplantation and either death, or last date of follow-up.

Statistical Analysis

IBM SPSS Statistics for Windows (IBM Corp. Released 2011. Version 20.0. Armonk, New York, USA) was used for data analysis. Results are presented as mean±SD, median, and range for continuous variables, and as numbers and percentages for categorical variables. To estimate the graft and patient survival rates, Kaplan-Meier survival analysis was used and comparison of graft survival rates was made using the log-rank test. Comparing different variables, independent-samples *Student’s t* test, Mann-Whitney U test, χ^2^ test, one-way ANOVA, and Kruskal-Wallis one-way analysis of variance were used when applicable. Cox proportional hazard model was used to investigate the associations between patient characteristics and the graft outcome. A p value <0.05 was considered statistically significant.

## RESULTS

A total of 1904 kidney transplantations was performed at Dr. Shariati Hospital, Tehran, Iran, during 1989–2013. Of these, 314 (16.5%) transplantations were done on pediatric recipients. The mean±SD follow-up period was 15.9±4.0 (range: 0.5–20) years. Table 1 demonstrates recipient, donor, and transplant characteristics.

**Table 1:** Recipient, donor, and transplant characteristics of pediatric kidney transplantation in Iran

**Variable**	**Statistics**
Recipient demographic characteristics	
Age, mean±SD (range), yrs	11.1±3.7 (3–18)
Age Group (yrs), n (%)	
3–6	31 (9.9)
7–10	127 (40.5)
11–14	89 (28.3)
15–18	67 (21.3)
Male sex, n (%)	164 (52.5)
Dialysis status before the transplantation, n (%)	
Pre-emptive transplantation	91 (29)
Hemodialysis	199 (63.4)
Peritoneal dialysis	24 (7.6)
Dialysis duration before the transplantation (yrs), n (%)	
<1	74 (33.2)
1–3	109 (48.9)
>3	40 (17.9)
Donor characteristics	
Age, mean±SD (range), yrs	34.7±8.3 (15–53)
Age group (yrs), n (%)	
<20	17 (5.4)
20–40	253 (80.6)
>40	44 (14)
Male sex, n (%)	213 (67.8)
Type, n (%)	
Living related	42 (13.4)
Living unrelated	251 (79.9)
Deceased brain-dead	21 (6.7)
Immunosuppressive regimen, n (%)	
Steroid + CSA + AZA	53 (16.9)
Steroid + CSA + MMF	144 (45.9)
Steroid + MMF + TAC	70 (22.3)
Steroid + MMF + SRL	47 (14.9)
Second transplantation, n (%)	17 (5.4)
Acute rejection episode, n (%)	
0	224 (71.3)
1	78 (24.8)
>1	12 (3.8)
Graft loss, n (%)	85 (27.1)
Malignancy, n (%)	9 (2.9)
Mortality, n (%)	11 (3.7)

AZA: azathioprine, CSA: cyclosporine, MMF: mycophenolate mofetil, SRL: sirolimus, TAC: tacrolimus

**Figure 1:**
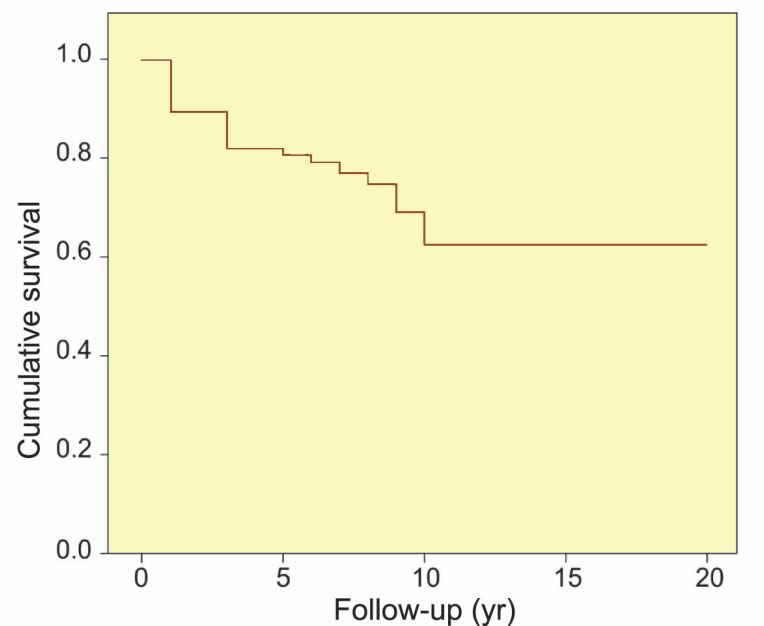
The overall graft survival among pediatric recipients of kidney transplantation

The mean±SD graft survival was 14.5±0.5 (range: 0.5–20; 95% CI: 13.4–15.5) years. The 1-, 3-, 5-, 10-, and 20-year graft survival rates (SEM) were 90% (2%), 82% (2%), 81% (2%), 62% (4%), and 62% (4%), respectively (Fig. 1). Although the mean±SD graft survival was higher among recipients aged 7–10 years, and also among girls (14.8±0.7 years; 95% CI: 13.3–16.1) than boys (11.8±0.5 years; 95% CI: 10.6–12.8), recipient age and sex were not significant determinants of graft survival (p=0.19 and p=0.34, respectively). The associations between graft survival rates and studied variables are summarized in Table 2.

**Table 2:** Graft survival rates at 1, 3, 5, and 10 years after kidney transplantation among pediatric patients based on different recipient, donor, and transplant characteristics

Variable	Graft survival rate (SEM), %				p value
	1-year	3-year	5-year	10-year	
Recipient age group (yrs)					0.19
3–6	80 (7)	80 (7)	80 (7)	40 (10)	
7–10	88 (2)	83 (3)	83 (3)	69 (6)	
11–14	96 (1)	81 (4)	76 (5)	76 (5)	
15–18	86 (4)	81 (4)	81 (4)	59 (8)	
Recipient sex					0.34
Male	85 (3)	77 (3)	77 (3)	64 (6)	
Female	94 (2)	87 (3)	85 (3)	62 (6)	
Dialysis status before the transplantation					0.006
Pre-emptive transplantation	91 (3)	86 (4)	86 (4)	86 (4)	
Hemodialysis	90 (2)	83 (3)	83 (3)	60 (6)	
Peritoneal dialysis	84 (8)	67 (11)	67 (11)	33 (14)	
Dialysis duration before the transplantation (yrs)					0.003
<1	87 (5)	58 (7)	58 (7)	29 (12)	
1–3	91 (3)	86 (4)	86 (4)	75 (6)	
>3	88 (6)	88 (6)	88 (6)	53 (11)	
Immunosuppressive regimen					0.001
Steroid + CSA + AZA	78 (5)	55 (6)	49 (6)	34 (7)	
Steroid + CSA + MMF	94 (2)	89 (3)	89 (3)	76 (7)	
Steroid + MMF + TAC	80 (6)	80 (6)	80 (6)	NA^*^	
Steroid + MMF + SRL	92 (3)	92 (3)	80 (5)	NA	
Presence of acute rejection					0.002
Yes	79 (4)	69 (4)	69 (4)	52 (7)	
No	93 (1)	87 (2)	85 (2)	66 (4)	
Donor type					0.002
Living related	100 (0)	100 (0)	100 (0)	100 (0)	
Living unrelated	88 (2)	80 (2)	79 (2)	60 (4)	
Deceased brain-dead	85 (8)	85 (8)	85 (8)	42 (17)	
Donor age group (yrs)					0.054
<20	72 (13)	72 (13)	72 (13)	72 (13)	
20–40	87 (2)	86 (2)	86 (2)	60 (7)	
>40	93 (3)	89 (4)	89 (4)	89 (4)	
Donor sex					0.078
Male	89 (2)	86 (2)	86 (3)	73 (5)	
Female	85 (4)	85 (4)	85 (4)	56 (10)	

AZA: azathioprine, CSA: cyclosporine, MMF: mycophenolate mofetil, NA: not applicable, SEM: standard error of the mean, SRL: sirolimus, TAC: tacrolimus

**Figure 2:**
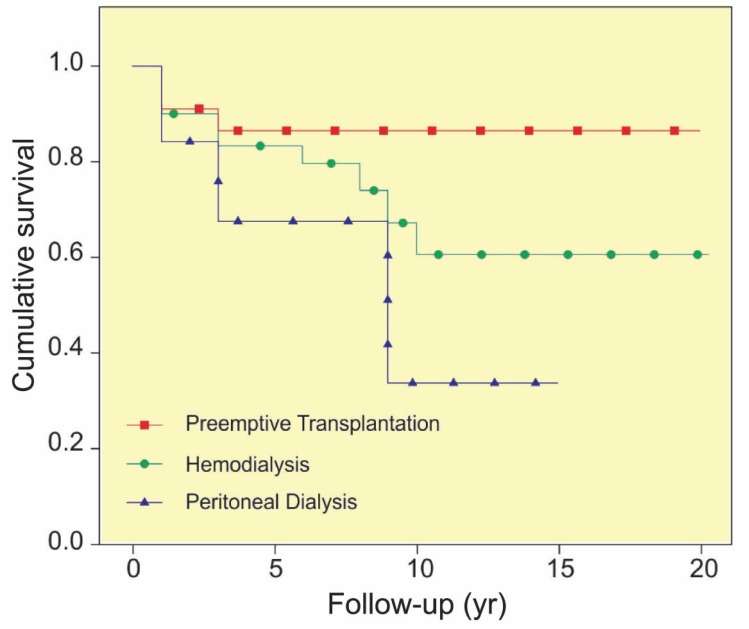
The graft survival among pediatric recipients of kidney transplantation based on the dialysis history before the transplantation (p=0.006

The mean±SD graft survival was significantly higher (p=0.006) among those who underwent pre-emptive transplantation (17.5±0.7 years; 95% CI: 16.0–19.0) than those who underwent hemodialysis (11.3±0.5 years; 95% CI: 10.3–12.3) or peritoneal dialysis (8.7±1.3 years; 95% CI: 6.1–11.4) before the transplantation (Fig. 2). Moreover, the mean±SD graft survival was significantly higher (p=0.003) among recipients who underwent the dialysis for 1–3 years (12.5±0.6 years; 95% CI: 11.2–13.8) than those who underwent dialysis for shorter periods (6.6±0.5 years; 95% CI: 5.5–7.6) or longer periods (10.8±1.1 years; 95% CI: 8.5–12.9), regardless of the type of dialysis.

**Figure 3:**
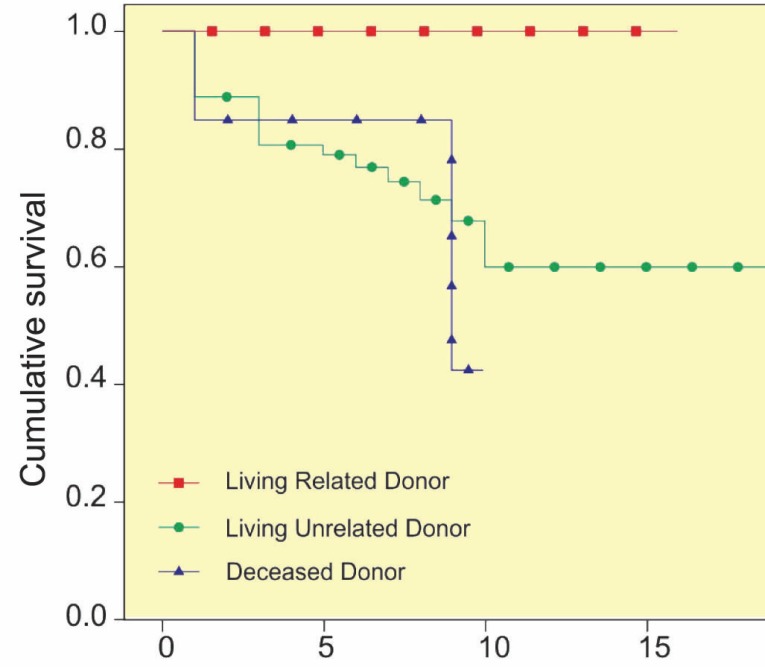
The graft survival among pediatric recipients of kidney transplantation based on donor type (p=0.002

The graft survival was higher among recipients of living donor grafts, and notably, as shown in Figure 3, recipients with a living-related donor had a 100% graft survival rate after 10 years that was significantly (p=0.002) higher than that in other groups. Although recipients of grafts from male donors and donors aged older than 40 years had higher graft survival rates, the differences were not statistically significant (p=0.078 and p=0.054, respectively).

Considering the immunosuppressive regimen, the recipients who received the combination of steroid plus cyclosporine plus azathioprine had a significantly (p=0.001) worse graft survival.

The causes of ESRD among the recipients were glomerular diseases (33.5%), congenital anomalies of the kidney and urinary tract (36%), tubulointerstitial diseases (12.5%), and unknown (18%). When looking at these causes, recipients with primary hyperoxaluria (PH) had the worst (p=0.001) and those with neurogenic bladder had the best graft survival rates (p=0.002). Other causes did not make significant differences regarding the graft survival. 

Seventeen patients underwent retransplantation. Surprisingly, these patients had a 10-year graft survival of 100% that was significantly (p=0.033) higher than others.

A total of 28.6% of the recipients experienced at least one episode of acute rejection and had a significant (p=0.002) lower mean±SD graft survival (9.4±0.6 years; 95% CI: 8.2–10.6) than those without any acute rejection episodes (15.3±0.6 years; 95% CI: 14.1–16.5). However, the mean±SD graft survival was lower among those with more than one acute rejection episodes (7.1±1.3 years; 95% CI: 4.4–9.7) compared to recipients with one episode (9.9±0.6 years; 95% CI: 8.6–11.2). The difference was not significant (p=0.28), however. 

Graft loss occurred following 27.1% of transplantations; the most common cause was chronic rejection that occurred in 37 (44%) recipients. A total of 13 (15%) patients experienced disease recurrence, mostly those with the primary diseases of focal segmental glomerulosclerosis (FSGS) and PH. The other causes of graft loss were acute rejection in 12.9%, discontinued immunosuppression due to noncompliance in 11.8%, renal vein thrombosis in 3.5%, and deaths with functioning graft in 12.9%.

The frequency of post-transplantation malignancy among our patients was 2.9%, including six recipients with lymphoproliferative diseases and three with other malignancies. These malignancies occurred after a median period of 4.5 years post-transplantation. The patient survival rate among these recipients was 100%. 

The 1-, 3-, 5-, 10-, and 20-year patient survival rates (SEM) among our recipients were 100% (0%), 100% (0%), 99.4% (1%), 97.8% (1%), and 96.5% (2%), respectively. Totally, 11 patients died during the follow-up period. The cause of death for all these patients was systemic infection that caused the death after a median period of 11.5 years post-transplantation. The mean±SD serum creatinine level of patients after 1, 3, 5, 10, and 20 years of transplantation was 1.04±0.20, 1.08±0.18, 1.14±0.30, 1.20±0.23, and 1.24±0.17 mg/dL, respectively.

Cox proportional hazard analysis revealed that dialysis history (HR: 1.14; 95% CI: 0.64–1.88; p=0.02), acute rejection (HR: 2.18; 95% CI: 1.63–2.80; p=0.001), and donor type (HR: 1.62; 95% CI: 1.27–2.12; p=0.01) were significant factors to predict the graft outcome.

## DISCUSSION

Kidney transplantation is the treatment of choice for pediatric patients with ESRD. However, a recent study in Iran revealed that only less than half of these children have the chance to undergo kidney transplantation [1]. Moreover, despite the improvements in the outcome of pediatric kidney transplantation in Iran, similar to developed countries, the long-term graft survival is not yet favorable [6, 11, 13, 14]. Therefore, it seems necessary to identify the factors that may influence the graft outcome, in order to improve the long-term graft survival in pediatric kidney transplantation.

Herein, we presented our experience in pediatric kidney transplantation over 25 years. Although a few studies have previously investigated the outcome of kidney transplantation among children in Iran [11, 13, 14], the current study is different from various aspects. Being a single-center study allows for better follow-up of patients, more detailed information, and longitudinal data, especially about the long-term outcomes of pediatric kidney transplantation. Also, the outcome of transplantation in our study is better than previous reports from Iran. A recent multi-center nationwide study in Iran shows that 1-, 3-, 5-, 10-, and 15-year graft survival rates are 88.7%, 80%, 72%, 59%, and 45%, respectively [13]. Furthermore, two previous single-center studies found 5-year graft survival rates of 56% and 67% [11, 14]. We found the graft survival rates were 90%, 82%, 81%, 62%, and 62% at 1, 3, 5, 10, and 20 years of the transplantation, respectively, comparable with and even better than the findings reported by 2010 annual transplant report of North American Pediatric Renal Trials and Collaborative Studies (NAPRTCS) [15]. 

More than half of our recipients were boys, compatible with previous studies in Iran and other countries [7, 10-16]. A previous study indicates that genetic factors and sociocultural beliefs might be the causes of higher frequency of kidney transplantation and CKD in boys [1, 17]. A recent study shows that graft survival is better among boys than girls [7]. In our study, although insignificant, the graft survival was better among girls, which is compatible with a previous study from Iran [11].

About two-thirds of our transplant patients were 7–14 years old. We did not have the experience of kidney transplantation in infants and very young children. It was previously indicated that pediatric kidney transplantation in developing countries is usually limited to older children [18]. However, the mean age of our patients was comparable with the mean age of pediatric patients with ESRD in Iran [1]. Some of the previous studies have reported that graft survival have been lower among adolescents [6, 7, 12]. Nevertheless, we found no significant difference in graft survival of recipients in different age groups, which was in keeping with the results of previous studies conducted in Iran [13].

It is generally accepted that pre-emptive transplantation should be considered in children when possible, and that the major criterion is the availability of a suitable donor [8, 19]. Meanwhile, some exclusion criteria have been proposed [8]. Moreover, it has been shown that graft survival is better in children who undergo pre-emptive kidney transplantation rather than those in whom transplantation is performed after a period of dialysis [7, 19, 20]. Our findings showed that among patients, the 29% who underwent pre-emptive transplantation had better graft survival. Other previous studies in Iran, and other countries have reported frequency of pre-emptive kidney transplantation among children to be 27% to 31% [11, 13, 14], and 8% to 52% [7, 10, 12, 15, 19-25], respectively. Based on our findings, among recipients who underwent dialysis before the transplantation, the graft survival was significantly higher in those who experienced dialysis between 1–3 years rather than those who underwent the dialysis for shorter or longer periods.

In different countries, the donor sources are different based on the religious, cultural, and ethnical variations [13]. Some countries prefer deceased donors [12, 16, 26]; some prefer living donors [19, 21], and some use both sources almost equally [7, 10, 15, 20, 25]. However, it is stated before that the graft source in developing countries is usually from living donors [18]. In this study, most of the grafts were from living donors, which was compatible with previous reports from Iran [11, 13, 14]. We have previously shown that graft survival is better when using living donor grafts rather than deceased donor ones among adult patients [27]. Our current findings confirmed that in addition to adults, using living donors led to better graft survival in children too, which is reported by some of the previous reports too [13, 23, 28-31]. Surprisingly, patients whose grafts were from living-related donors had a graft survival rate of 100% after 10 years. Regarding the donor age and sex, we did not find any significant differences in graft survival, though some previous studies demonstrate that graft survival is worse with older-aged donors [10, 13]. However, it is also indicated by previous studies that except for very young pediatric recipients, the graft-size mismatch is not an important issue and generally, size and age matching is not required in kidney transplantation [6].

All of our patients received steroid as part of their immunosuppressive therapy. The most frequent combination immunosuppressive regimen among our patients was steroid plus cyclosporine plus mycophenolate mofetil. Our patients received the drug combinations based on the availability and applicability at the time of transplantation. Different studies have reported the use of different combinations of immunosuppressive agents [20, 32]. It is also demonstrated that steroids, cyclosporine, and azathioprine are the mainstay of immunosuppression in developing countries [18]. However, the combination of these three agents led to the worst graft survival among our recipients.

It is reported previously that the most common causes of ESRD among children are congenital or inherited anomalies, and glomerulonephritis [6, 8]. A recent study investigating the incidence and etiologies of CKD among Iranian children found glomerular diseases as the leading cause of ESRD among pediatric patients, followed by congenital anomalies of kidney and urinary tract [1]. However, different studies have reported different leading causes [13, 15, 16, 19, 21-23, 26, 32]. Among our patients, the most common etiologies of ESRD were congenital anomalies of kidney and urinary tract, followed by glomerular diseases. Although some studies have found no differences between ESRD etiologies regarding the graft survival [10], a recent study reports that patients with an ESRD etiology of FSGS have lower graft survival [7]. In the current study, we found that patients with an etiology of PH and neurogenic bladder had, respectively, the worst and the best graft survivals, while other etiologies were not significantly different. We believe that lower graft survival of patients with PH was due to unavailability of combined liver-kidney transplantation in the past in Iran, as when we performed the first sequential liver-kidney transplantation for a child with type 1 PH for the first time in Iran, the results were favorable [33-35].

Different studies have reported various rates of retransplantation based on their study design and transplantation outcome [12, 13, 15, 16]. In the current study, 5.4% of our patients experienced a second kidney transplantation. Surprisingly, the graft survival was significantly better among these patients. This could be due to different reasons—better adherence to medications, newer and more potent immunosuppressive drugs, and the overall improvement of kidney transplantation outcome over the time in Iran.

Previous studies have demonstrated that episodes of acute rejection are associated with poor long-term survival rates [10, 13, 14, 36]. Therefore, the graft survival could be improved by further decrease in the incidence of acute rejection [10, 37]. Meanwhile, the rates of acute rejection episodes have decreased among children over the time [12, 14, 15]. A previous study from Iran reports that acute rejection occurs in 39.5% of children [13]. Furthermore, based on the 2010 annual transplant report of NAPRTCS, 45.6% of pediatric patients in the USA experience at least one episode of acute rejection, while among about half of them it occurs more than once [10]. Other studies have reported rates between 15% and 39% [10, 20-22, 26, 32]. In the current study, 28.6% of our patients experienced at least one episode of acute rejection. These patients had significantly lower graft survival than those without acute rejection. In addition, most of these patients experienced only one episode of acute rejection. The differences in the frequency of acute rejection in various studies could be due to different causes. One important factor is the definition of acute rejection in the study design and the diagnostic method used, whether clinically or by biopsy. Other causes might be the different sources of donors, and immunosuppression. However, efforts should be made to decrease the incidence of acute rejection to improve the graft survival among children.

Similar to previous reports, chronic rejection was the most common cause of graft loss among our patients [12, 13, 15]. Besides, disease recurrence occurred in about 15% of our recipients, which was more than that reported earlier [12, 13, 15]. Most of the disease recurrences happened in patients with primary diseases of FSGS and PH.

Children are at increased risk for developing malignancies following kidney transplantation compared with adults and the general population [8, 38]. A previous study in Iran reports the post-transplantation malignancies to occur in only 0.6% of pediatric recipients [13]. This rate is 2.36% and 7.3% among children of the USA and France, respectively [12, 15]. In the current study we found a prevalence rate of 2.9%, two-thirds to be lymphoproliferative and the other one third to be non-lymphoproliferative disorders. It has been estimated that with the current use of more potent immunosuppressive agents, the prevalence of malignancies after pediatric transplantation might become higher in the future [12]. Although previous studies demonstrate malignancies as one of the major causes of death among pediatric recipients, none of our patients died of malignancies [12, 23].

Many studies report various predicting factors of graft survival including recipient age, sex, and race, donor age and type, dialysis history before the transplantation, previous transplant history, transplant year, acute rejection, delayed graft function, and GFR [10, 12, 13, 15, 20, 23]. However, we found only acute rejection, dialysis history, and donor type as independent predictors of the graft survival.

It is stated before that kidney transplantation allows more than 90% of pediatric patients to survive for at least 20 years [29]. The patient survival after kidney transplantation has improved over the time and a patient survival more than 85% at 10–20 years after transplantation should be reasonably expected [7, 12]. Previous studies report post-transplantation infections and malignancies as the major causes of death among children [6, 12, 15, 18, 39]. They mention different mortality rates between 3% and 27% [12, 13, 15, 16, 39-42]. Also regarding the patient survival rate, previous studies report 1-, 5-, 10-, and 20-year rates of 92%–100%, 85%–98%, 69%–97%, and 84%, respectively [7, 11, 12, 14-16, 21, 23, 26, 36, 40, 41]. The mortality rate among our patients was 3.7%. All of these recipients died of post-transplantation systemic infections, resulting in 1-, 5-, 10-, and 20-year patient survival rates of 100%, 99.4%, 97.8%, and 96.5%, respectively, which were higher than the above-mentioned figures. However, as mentioned earlier, patient survival in pediatric kidney transplantation surpasses the graft survival that necessitates further efforts to improve graft survival in the future [7].

Our study was limited in some aspects. As this was a retrospective study over 25 years, we could not have complete data about some variables such as growth, HLA mismatch, panel reactive antibody test results prior to transplantation, detailed types of graft rejections, and delayed graft function status, so that we had to eliminate these variables in our analysis. Furthermore, although we provided overall 20-year graft and patient survival rates, we could not investigate the associations of different variables with graft survival at 20 years of transplantation, because many of the studied patients have not yet reached the 20-year milestone. We did also not investigate the graft outcome in different eras, as it was frequently performed before [7, 10, 12, 13, 15, 16]. In spite of these limitations, we consider our cohort study over 25 years one of the largest single-center investigations in pediatric kidney transplantation that can provide noticeable information regarding the long-term graft survival among children. This would be more important when we note the findings of graft outcome in our study were comparable to those of pediatric kidney transplantation in the USA-based 2010 annual transplant report of NAPRTCS [15], taking into account that we are in a developing country with limited access to newer and more potent immunosuppressive agents.

In conclusion, the main findings of the current study were as follows: (1) the 20-year graft and patient survival rates were 62% and 96.5%, respectively; (2) pre-emptive transplantation and living kidney donation were positive factors in graft survival; (3) acute rejection and immunosuppressive regimen of steroid plus cyclosporine plus azathioprine were associated with lower graft survival; (4) acute rejection, dialysis history before the transplantation, and donor type were the significant predictors of long-term graft outcome among children; (5) chronic rejection was the most common cause of graft loss; (6) patient survival yet surpasses the graft survival in pediatric kidney transplantation; (7) short-term graft survival still outpaces the long-term outcome in the pediatric kidney transplantation; (8) still greater improvements in graft survival, particularly long-term, are needed; (9) as the effective factors on graft survival are modifiable, with more intense immunosuppression for greater prevention of acute and chronic rejection, and increased rate of pre-emptive and living donor transplantation, long-term graft survival may significantly improve among children in the near future.
